# Nucleolar origins: challenging perspectives on evolution and function

**DOI:** 10.1098/rsob.240330

**Published:** 2025-03-12

**Authors:** Israel Muñoz-Velasco, Ana Karen Herrera-Escamilla, Alberto Vázquez-Salazar

**Affiliations:** ^1^ Departamento de Biología Celular, Universidad Nacional Autónoma de México, Ciudad de México, México; ^2^ Department of Chemical and Biomolecular Engineering, University of California Los Angeles, Los Angeles, CA, USA

**Keywords:** nucleolus, nucleolus-like structures, nucleolus evolution, nucleolar organization

## Abstract

The nucleolus, once considered a mere ‘ribosome factory’, is now recognized as a dynamic hub influencing nearly every aspect of cellular life, from genome organization to stress response and ageing. Despite being a hallmark of eukaryotic cells, recent discoveries reveal that even prokaryotes exhibit nucleolus-like structures, hinting at ancient origins for nucleolar functions. This review explores the evolutionary journey of the nucleolus, tracing its roots back to early life and examining its structural and functional diversity across domains. We highlight key nucleolar proteins that play vital roles not only in ribosome production but also in regulating cell cycle, DNA repair and cellular stress, linking nucleolar activity directly to health and disease. Dysfunctions in nucleolar processes are implicated in cancer, ribosomopathies and neurodegenerative disorders, positioning the nucleolus as a critical target for innovative therapeutic strategies. As advanced imaging and molecular techniques unlock deeper insights into both canonical and mysterious non-canonical roles, the nucleolus stands as a model for how cellular microenvironments can evolve to meet complex biological demands. By addressing open questions surrounding the evolution of the nucleolus, its organization and diverse functions, the ideas presented here aim to contribute to the ongoing discussion, challenging traditional paradigms and suggesting new avenues for uncovering the fundamental principles that drive cellular life.

**Figure:**
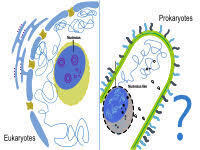

## 1. The nucleolus: a cellular powerhouse

**Figure 1.**
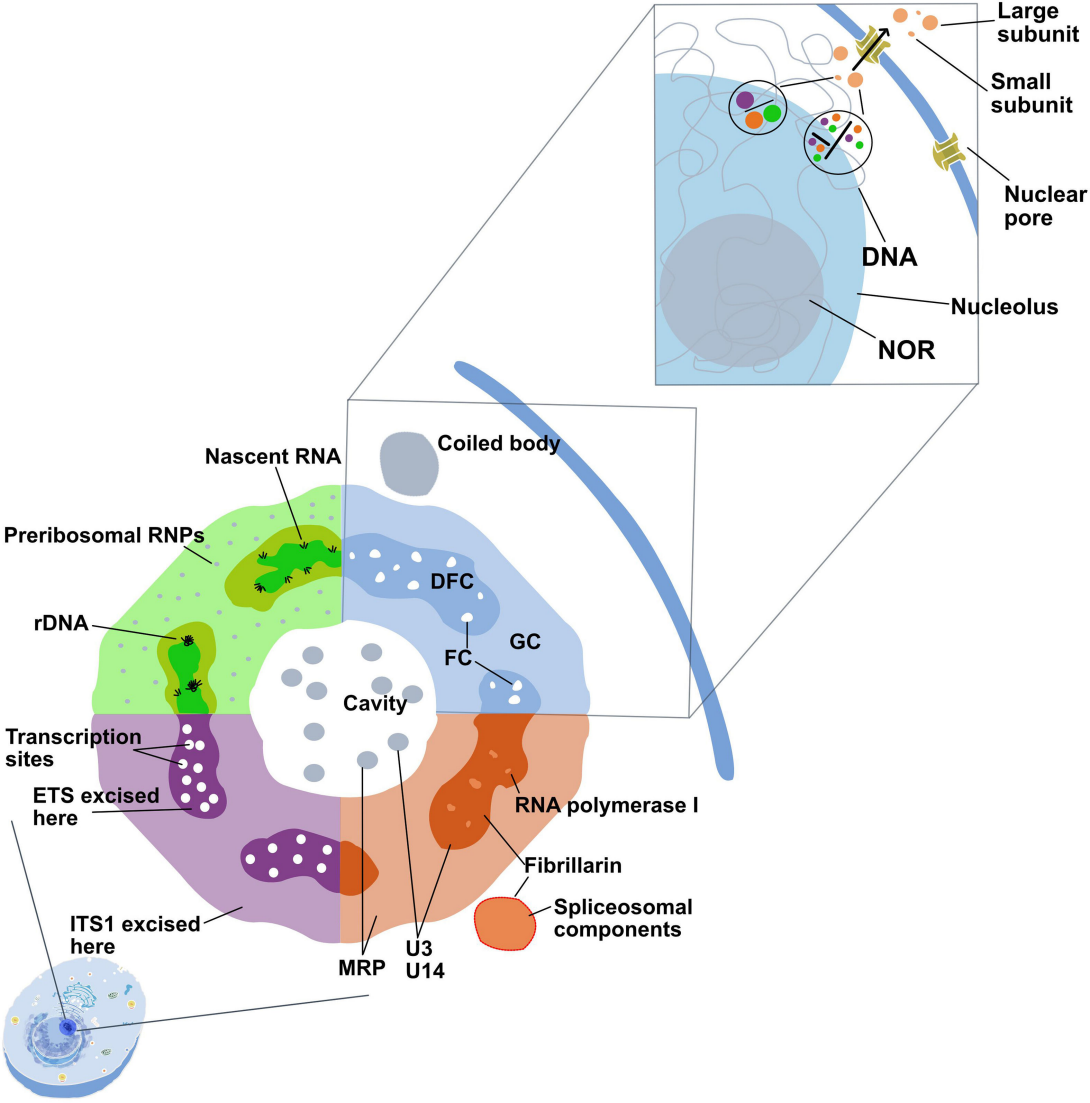
Detailed structure of the eukaryotic nucleolus. This schematic illustrates the complex organization of the nucleolus, highlighting distinct regions and functions. Key compartments include the fibrillar centre (FC), dense fibrillar component (DFC) and granular component (GC). The nucleolus contains transcription sites where rDNA is transcribed, generating nascent RNA, which is then processed and assembled with ribosomal proteins in the granular component to form preribosomal RNPs. Additional elements such as the coiled body, RNA polymerase I and spliceosomal components are present, illustrating the role of the nucleolus in ribosome biogenesis and other cellular processes, Image inspired by [1, fig. 4].

The nucleolus is a membrane-less, highly regulated substructure within the nucleus, formed around ribosomal RNA gene clusters known as nucleolar organizer regions (NORs) (figure 1) [2]⁠. It plays a critical role in ribosomal RNA (rRNA) synthesis, overseeing the transcription of rRNA genes, processing precursor rRNAs and assembling ribosomal subunits essential for ribosome biogenesis in eukaryotic cells [3,4]⁠⁠. Beyond its primary role in ribosome production, the nucleolus is involved in regulating the cell cycle, responding to cellular stress and influencing ageing [5]. It also facilitates the assembly of signal recognition particles for protein synthesis and modifies small nuclear RNAs (snRNAs), which are essential for gene expression [6]. Additionally, the nucleolus sequesters proteins involved in cellular metabolism and gene regulation, serving as a hub for the assembly and modification of these essential cellular components [7]⁠.

Alterations in nucleolar structure and function have been associated with various diseases, including cancer, neurodegenerative disorders and viral infections [8,9]⁠. These links highlight the nucleolus as a critical player in cellular regulation and a promising target for therapeutic interventions [10]⁠. A deeper understanding of its diverse functions is essential to uncovering its broader implications in cellular biology and disease.

### 1.1. Understanding the known aspects of the nucleolus is essential to identify the unknown

While its role in ribosome production is well-established, its broader involvement in cellular processes remains less clear. This knowledge gap highlights the need for a deeper exploration of nucleolar structure, functions and dynamics to fully grasp its impact on cellular physiology (figure 1), studying the evolution of the nucleolus is crucial for understanding the complexity of ribosomal biogenesis and cellular function across diverse organisms across the tree of life. Although bacteria and archaea lack subcellular structures like those in eukarya, they rely on analogous ‘nucleolus-like’ mechanisms for essential functions. The main tasks of the ‘nucleolus-like’ structure are essential for the correct function of those organisms.

The nucleolus has undergone significant evolutionary adaptations, reflecting selection pressures and changes in cellular demands [11]⁠. Gaining insight into fundamental nucleolar processes not only sheds light on cellular function but also provides valuable perspectives on diseases linked to nucleolar dysfunction [12]⁠.

**Figure 2.**
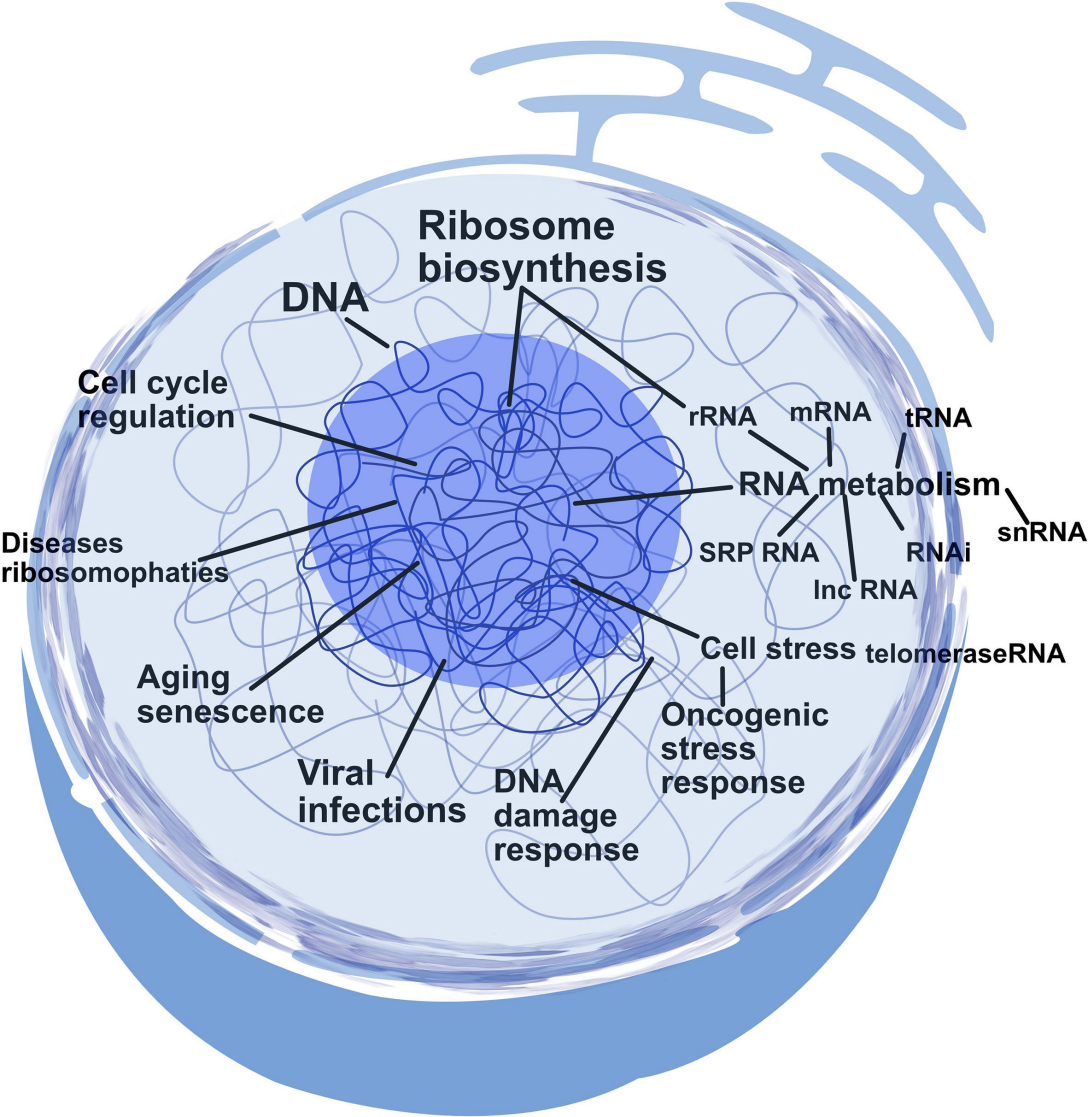
Functional roles of the nucleolus in cellular processes In addition to ribosome production, the nucleolus is involved in regulating cell cycle progression, responding to DNA damage and managing cellular stress. It also plays a role in ageing, viral infections, oncogenic stress response and RNA metabolism. The diagram reflects how the nucleolus integrates signals related to cell growth, repair and stress response, underscoring its importance in both normal cellular function and disease.

Nucleolar dysfunction is associated with various human diseases, including cancer and neurodegenerative disorders [13]⁠. Disruptions in nucleolar structure and function can impair ribosome biogenesis, destabilizing cellular homeostasis and contributing to disease progression [9]⁠ (figure 2). By deepening our understanding of nucleolar function, we can uncover how these dysfunctions influence disease, potentially paving the way for novel diagnostic and therapeutic strategies. This potential for innovative treatments and diagnostic tools is a beacon of hope and inspiration in modern cellular and molecular biology.

Recent advancements in imaging, molecular biology techniques and proteomic approaches have provided unprecedented insight into nucleolar dynamics and functions, revealing previously unexplored areas [14]⁠. Super-resolution microscopy has allowed detailed visualization of nucleolar architecture and its changes under different physiological conditions [15]⁠. Techniques like CRISPR-Cas9 genome editing enable precise manipulation of nucleolar components, facilitating the study of their specific roles [16]⁠. Meanwhile, proteomic analyses have identified over 1000 proteins involved in nucleolar processes, many of which remain uncharacterized [17]⁠. These innovations have significantly enhanced our understanding of the nucleolus and its role in cellular biology.

Continual exploration of nucleolar biology is essential for uncovering new dimensions of its significance and potential applications. Despite being a long-standing subject of scientific inquiry, this enigmatic nuclear subcompartment continues to captivate researchers with its multifaceted nature and the wealth of unanswered questions regarding its structure, function and regulation. The complexity and mystery of the nucleolus continue to inspire further research, driving the field of cellular and molecular biology forward.

## 2. Structural complexity of the nucleolus: morphological and functional insights

As our understanding of nucleolar architecture has evolved, several key substructures have emerged as central to its function (figure 1). The fibrillar centre (FC) is one such critical region, serving as the site where ribosomal DNA (rDNA) is housed. While the FC contains inactive copies of rDNA, transcription itself primarily occurs at the interface between the FC and the surrounding dense fibrillar component (DFC), where RNA polymerase I complexes initiate the synthesis of pre-45S rRNA [18]⁠. This central hub provides a scaffold for the transcription machinery and sets the stage for ribosome production.

The dense fibrillar component (DFC) plays an instrumental role in processing pre-45S rRNA into the mature 18S, 5.8S and 28S rRNA segments, which are key building blocks of eukaryotic ribosomes. Additionally, important modifications, such as methylation and pseudouridylation, occur within the DFC, preparing the rRNAs for their eventual integration into ribosomal subunits. The DFC also facilitates the assembly of ribosomal proteins, imported from the cytoplasm, with the processed rRNAs to ensure the proper maturation of ribosomal subunits. Its interaction with the granular component (GC) enables a coordinated progression from rRNA processing to ribosome assembly [19]⁠.

The granular component (GC), which surrounds the DFC, is essential for the final stages of ribosome biogenesis. Here, pre-ribosomal particles undergo maturation and assembly, with proteins such as nucleophosmin (B23) playing key roles in this process. The size of the GC is directly linked to the metabolic activity of the cell, reflecting the demand for ribosome production during growth and division [20]⁠. The continuous collaboration between the GC, DFC and FC ensures the seamless synthesis and maturation of functional ribosomes, which are essential for cellular homeostasis.

In addition to these well-characterized regions, recent discoveries have identified less-understood nucleolar substructures. For example, nucleolar ‘vacuoles’ (NoVs), which are membrane-less structures observed primarily in plants and some animals like *Caenorhabditis elegans*, represent areas with fewer granules and fibrils. Their exact functions, especially in relation to ageing in germline cells, remain largely speculative, but their association with FCs suggests a potentially important role in nucleolar dynamics [21]⁠.

A more recently discovered substructure is the nucleolar rim (NR), a distinct compartment characterized by its unique proteomic composition. The proteins within the NR are notably more disordered compared to other nucleolar proteins, raising intriguing questions about their function. The NR is thought to tether the nucleolus to chromatin, potentially influencing processes such as cell cycle regulation and ribosome biogenesis, though this remains an active area of investigation [17]⁠.

Further adding to the complexity of the nucleolar landscape, recent studies have revealed the presence of physicochemical ‘barcodes’ within the nucleolus. These barcodes reflect distinct subcompartments that create unique microenvironments characterized by varying electrical, electrochemical, mechanical, hydrodynamic and biochemical properties [22]⁠. Understanding these microenvironments could unveil additional layers of nucleolar function and regulation, offering new perspectives on how the nucleolus adapts to the diverse demands of the cell.

Together, these substructures and emerging discoveries underscore the intricate organization and its ability of the nucleolus to dynamically respond to cellular needs. As research continues to uncover new aspects of nucleolar architecture, it becomes increasingly clear that each component plays a vital role in maintaining cellular function and supporting essential biological processes.

### 2.1. Communication and interaction between nucleolus and nuclear regions

The nucleolus not only drives ribosome biogenesis but also plays a significant role in organizing the genome within the nucleus. It interacts with chromatin through nucleolar-associated domains (NADs), which are regions of the genome characterized by low transcriptional activity and high heterochromatin density near the nucleolus [23]⁠. These NAD domains are typically enriched with H3K9me2 histone modifications, markers commonly associated with transcriptional repression. By organizing these repressive chromatin regions, the nucleolus contributes to the overall architecture of the nucleus, which is divided into areas of euchromatin (active) and heterochromatin (inactive). The nucleolus is often seen as a hub for the organization of inactive chromatin, influencing gene expression patterns and maintaining genomic stability [24]⁠.

Techniques like nucleolus Hi-C (nHi-C) have been used to better map the interactions between nucleolus-associated chromatin and the rest of the genome. This method allows the capture of nucleolus-specific chromatin interactions, revealing that a significant portion of the genome forms high-confidence nucleolus-associated domains (hNADs) that cluster around nucleolar organizer regions (NORs) and centromeres. These interactions are essential for maintaining the structural integrity of chromatin and regulating transcriptional activity [23]⁠.

The nucleolus serves as a core centre that influences the spatial arrangement and function of other nuclear structures. For example, disrupting nucleolar structure by knocking down specific genes and, therefore, down protein expression can lead to significant changes in the spatial organization of nuclear bodies such as Cajal bodies. These structures are involved in the maturation and modification of small nuclear RNAs (snRNAs) and small nucleolar RNAs (snoRNAs). Cajal bodies are also essential for ribosome biogenesis, telomere maintenance, gene regulation, nuclear stress response and RNA splicing [25]⁠.

Nucleolar proteins frequently shuttle between the nucleolus and the nucleoplasm, especially in response to cellular stress. Under conditions such as DNA damage, oxidative stress or when ribosome production is inhibited by treatments like Actinomycin D, proteins involved in ribosome biogenesis, such as nucleophosmin (NPM1), are translocated from the nucleolus to the nucleoplasm. This translocation triggers stress response pathways, including the activation of the p53 signalling pathway, a critical regulator of the cellular stress response [8]⁠.

The dynamic movement of these proteins underscores the direct communication between the nucleolus and the broader nuclear environment. Maintaining nucleolar structural integrity is vital for its function in sensing and responding to stress. Disruptions in nucleolar structure not only affect ribosome biogenesis but also lead to abnormal nucleolar morphology, impaired signalling and contribute to cellular dysfunction [26]⁠.

## 3. The nucleolus’s cast of characters: proteins and their roles

### 3.1. Overview of some essential proteins found in the nucleolus and their functions

Understanding the diverse array of proteins in the nucleolus is crucial for revealing its functions and dynamics [18]⁠. Advances in proteomics have significantly improved our ability to identify and characterize the proteins in this subnuclear structure. The nucleolus contains both ribosomal proteins and enzymes involved in ribosome biogenesis, as well as a wide range of non-ribosomal proteins that contribute to processes like DNA repair, RNA processing, chromatin remodelling, transcription regulation and cell death [18,27]⁠. Comparative studies have shown that the composition and organization of the nucleolus can vary among species. For example, yeast nucleoli lack certain proteins found in higher Eukaryotes, reflecting evolutionary divergence in nucleolar function [28]⁠. While over 40 nucleolar proteins have been identified (table 1), some of the most extensively studied include ribosomal proteins, RNA polymerase I, nucleophosmin (NPM1), fibrillarin and nucleolin. Ongoing research continues to uncover new proteins and their functions within the nucleolus. For instance, nucleostemin and nucleolar protein 1 (NOL1/NOP2/Sun domain family, member 2) are involved in stem cell maintenance, nucleolar stress responses and regulation of ribosome biogenesis [82]⁠. Nucleolar protein 4 (NOL4), implicated in DNA repair, cell proliferation and metastasis, has emerged as a potential target for cancer immunotherapy [83]⁠.

**Table 1.** Key nucleolar proteins, their functions and evidence of nucleolar association.

protein name	function	evidence of relationship with the nucleolus	references
NPM1 (Nucleophosmin	participating in ribosome biogenesis, mRNA processing, chromatin remodelling, embryogenesis, cell cycle regulation and apoptosis	important in nucleolar function and ribosomal biogenesis	[29]
nucleolin	rDNA transcription, rRNA maturation, ribosome assembly, nucleocytoplasmic transport and apoptosis regulation	abundant protein in the nucleolus is involved in RNA processing	[30]
fibrillarin	involved in pre-rRNA methylation and processing and pre-ribosome assembly	protein identified in proteomic analyses of nucleoli	[31]
nucleostemin	interaction with p53; plays an indispensable role in early embryogenesis, cell growth regulation, the self-renewal of stem or progenitor cells and ribosome biogenesis	localized in the nucleolus, unrelated to ribosomal biogenesis	[32]
HSP70	chaperone, folding of newly synthesized proteins, the translocation of polypeptides into mitochondria, chloroplasts and the endoplasmic reticulum (ER), disassembling protein complexes and regulating protein activity	detected in the nucleolus, involved in cellular stress responses	[33]
RPA (DNA binding protein)	involved in DNA repair, replication and recombination	participates in DNA damage response in the nucleolus	[34]
p53	regulates biological processes like the stress response, cell cycle, proliferation, invasion, senescence, apoptosis and autophagy	associated with nucleostemin in the nucleolus	[35]
cajal bodies proteins	RNA processing and modification are involved in the biogenesis of small ribonucleoproteins (RNPs), snRNA transcription and modification of snRNP assembly	associated with Cajal bodies within the nucleolus	[36,37]
SRP (signal recognition particle)	protein assembly and transport proper biogenesis of membrane and secretory proteins	involved in the localization of proteins in the nucleolus	[38]
TERC (telomerase RNA component)	structural scaffold for telomerase complex assembly	transiently visits the nucleolus	[39]
SIRT1	mediate the deacetylation of histones and non-histone proteins in an NAD^+^-dependent manner; transcription regulation and metabolism	associated with gene expression regulation in the nucleolus	[40]
cdk2	cell-cycle control, regulation through phosphorylation of the terminal tail of RNA polymerase II, metabolism and, in specific cell types, differentiation	implicated in cell cycle regulation in the nucleolus	[41]
PML	coordinates the assembly of nuclear aggregates named PML nuclear bodies (PML-NBs)	associated with nucleolar function in cellular regulation	[42]
UBF (upstream binding factor)	the rRNA production regulates rDNA transcription in response to growth factors and cell-cycle progression and replicates several viruses	involved in the transcription of nucleolar genes	[43]
BAF (barrier-to-autointegration factor)	nuclear structure regulation protects genome integrity and ensures the successful completion of mitosis	associated with nucleolar organization	[44]
PRMT5	protein methylation; methylate, both histone and non-histone proteins. Maintenance of tissue homeostasis as well as disease phenotypes	participates in the modification of nucleolar proteins	[45]
SRSF1	regulates post-transcriptional gene expression via pre-mRNA alternative splicing, mRNA stability, translation and regulator of mRNA metabolism	functions in the nucleolus in RNA processing	[46,47]
hnRNPs (heterogeneous nuclear ribonucleoproteins)	they are implicated in RNA metabolism, such as alternative splicing, mRNA stabilization and translational regulation; essential in nucleic acids metabolism and function, regulatory factors in stem cell potency and differentiation	they are involved in RNA processing in the nucleolus	[48,49]
TAF15	FUT1 mRNA stability plays a vital role in several key inflammation signalling pathways by maintaining target mRNA stability by regulating mRNA transcription, splicing and trafficking	it is associated with gene expression regulation	[50,51]
cdc2	cell cycle regulation is involved in binding cyclins A and B	implicated in mitosis regulation in the nucleolus	[52]
DDX21	ribosomal RNA processing and RNA polymerase II (RNA PolI)-mediated transcription, ribosome biogenesis and general transcription, sensing of cellular glucose levels for epidermal differentiation	it is associated with RNA processing in the nucleolus	[53,54]
NUP153	nuclear pore complex component transport, whereas the remainder of the protein maintains pore integrity and is essential for nuclear translocation	participates in nuclear transport regulation	[55,56]
p300	histone acetylation, transcription regulation and transcriptional coactivators bridge DNA-binding transcription factors to components of the basal transcriptional machinery	associated with gene expression regulation	[57]
TAF4	the transcription complex component regulates gene expression	participates in transcription regulation in the nucleolus	[58]
CTCF	chromatin structure regulation and transcriptional regulator	involved in nucleolar organization	[59]
CENP-A	necessary and sufficient for centromere specification and function, functional centromere maintenance	associated with nuclear organization	[60]
RUVBL1	chromatin remodelling, fanconi anaemia (FA), nonsense-mediated mRNA decay (NMD) and assembly and maturation of several large macromolecular complexes such as RNA polymerases, the box C/D small nucleolar ribonucleoprotein (snoRNP) and mTOR complexes	participates in nucleolar structure regulation	[61]
SMC1	the cohesin complex component of sister chromatid cohesion and DNA repair	associated with DNA organization in the nucleolus	[62,63]
RAD51	DNA repair, DNA double-strand (dsDNA) break repair by homologous recombination, protection of newly replicated DNA from nucleolytic degradation, homology recognition and DNA strand exchange	participates in DNA repair in the nucleolus	[64,65]
WRAP53	regulation of p53 expression, a scaffolding protein important for telomerase localization, telomere assembly, Cajal body integrity and DNA double-strand break repair	associated with cell cycle regulation in the nucleolus	[66]
HSF1	mediates downstream heat shock proteins (HSPs) expression at the transcriptional level to support cellular protein homeostasis by facilitating nascent protein synthesis, folding and degradation	involved in stress response in the nucleolus	[67]
GTPBP1	ribosome biogenesis participation, mRNA surveillance and ribosome-associated quality control	associated with RNA processing in the nucleolus	[68]
PML-RARA	transcription factors that deregulate transcriptional programs block the differentiation of hematopoietic progenitor cells, thus causing leukaemia	associated with nucleolar disrupted gene regulation in acute promyelocytic leukaemia	[69]
MDM2	E3 ubiquitin ligase degrades p53, a regulator of p53 that regulates several cellular processes, including cell-cycle control, apoptosis, differentiation, genome stability and transcription	associated with cell cycle regulation in the nucleolus	[70,71]
SIRT6	deacetylase, mono-ADP-ribosyltransferase and long fatty deacetylase participate in various cellular signalling pathways, from DNA damage repair in the early stage to disease progression	participates in gene expression regulation in the nucleolus	[72]
TERT	control telomerase activity	transiently visits the nucleolus	[73]
U2AF	mRNA processing, defining functional 3′ splice sites in pre-mRNA splicing	involved in RNA processing in the nucleolus	[74]
SRSF3	SRSF3 regulates constitutive and alternative splicing and additional aspects of RNA metabolism, such as alternative polyadenylation, mRNA export, transcription termination and miRNA biogenesis	it is associated with RNA processing in the nucleolus	[75]
HSF2	stress response regulation is involved in the onset of HSPs' expression, regulates (inhibits) their expression or controls the expression of other developmental genes	involved in stress response in the nucleolus	[76]
CENP-B	centromere structure regulation shapes the centromeric chromatin state	it is associated with nuclear organization	[77]
GADD45	DNA damage response, DNA repair, cell-cycle arrest and apoptosis	participates in DNA repair in the nucleolus	[78]
RPL11	it inhibits its ubiquitin ligase activity, coordinates the p53 response to nucleolar stress and activates p53 under oncogenic and replicative stresses	associated with gene expression regulation in the nucleolus	[79]
NOL1 (Nucleolar Protein 1)	regulation of ribosome biogenesis and cell growth and protein production	involved in nucleolar organization and function	[80]
nucleophosmin-like 1	ribosome biogenesis, DNA repair, genomic stability, molecular chaperoning, regulation of apoptosis and cell cycle regulation	it is associated with nucleolar function and stress responses	[81]

Both nucleophosmin (NPM1) and nucleolin play central roles in ribosome biogenesis, but their functions extend beyond this process.

NPM1 regulates cell death pathways in response to cellular stress and DNA damage, specifically during apoptosis, where it is involved in both intrinsic and extrinsic apoptotic pathways. Mutations in NPM1, such as those found in acute myeloid leukaemia (AML), lead to its mislocalization, disrupting its ability to regulate apoptosis [84,85]⁠⁠. Additionally, NPM1 acts as a chaperone of proteins involved in chromatin remodelling and DNA repair mechanisms [86]⁠ and interacts with tumour suppressor factors like ARF [87]⁠.

Nucleolin is essential for cell growth and proliferation, regulating gene expression through interaction with nucleic acids involved in transcription and translation [88]⁠. Interestingly, nucleolin also facilitates viral replication by interacting with viral proteins, highlighting its role in viral pathogenesis [89]⁠. Fibrillarin (FBL), a key player in rRNA methylation, is involved in the 2′-O-methylation of numerous pre-rRNA sites, a process crucial for ribosome biogenesis. As a core component of small nucleolar ribonucleoprotein (snoRNP) complexes, fibrillarin is vital for rRNA maturation and initiation of transcription by RNA polymerase I. Fibrillarin can undergo liquid–liquid phase separation (LLPS), facilitating the formation of membrane-less organelles like nucleolus [90]⁠. This protein also interacts with p53 and ARF, suggesting a potential role in cancer progression [91]⁠. Importantly, NPM1, nucleolin and fibrillarin all contain intrinsically disordered regions, which may regulate their localization and function within the nucleolus.

Recent studies have shown the presence of telomerase components in the nucleolus, suggesting that part of telomerase biosynthesis may occur there. This discovery has implications for cellular ageing and genomic instability, suggesting they influence telomere maintenance. Moreover, some nucleolar proteins re-localize to the chromosomal periphery during mitosis, indicating a role in nucleolar disassembly and reassembly during cell division. These proteins can be classified into two groups: those recruited early during prometaphase and those recruited later during post-metaphase. Understanding these recruitment dynamics helps clarify how the nucleolus disassembles and reassembles during the cell cycle.

## 4. A dynamic framework for nucleolar organization: liquid–liquid phase separation

Within the cell, certain biomolecules can undergo LLPS to form distinct, liquid-like compartments and facilitates the formation of dense, dynamic, liquid-like compartments not enclosed by membranes; the nucleolus is a prime example of a membrane-less organelle assembled through this process. LLPS is driven by interactions among intrinsically disordered regions (IDRs) in proteins and RNA molecules, which promote the formation of dynamic, non-membrane-bound compartments [92]. In the nucleolus, LLPS underlies the development of its well-defined subcompartments, the fibrillar centre (FC), dense fibrillar component (DFC) and granular component (GC), as described in §2. For instance, the DFC forms a phase-separated environment that concentrates rRNA processing factors, thereby enhancing the efficiency of ribosome biogenesis [93]. Similarly, the GC, which facilitates the final stages of ribosome assembly, arises through LLPS mediated by proteins such as nucleophosmin, whose IDRs promote the formation of liquid droplets [94,95]. These phase-separated compartments are not static; they exhibit fluid-like properties, allowing for the dynamic exchange of components and rapid adaptation to cellular needs. In essence, the nucleolus functions as a multiphase condensate, with each subcompartment representing a distinct liquid phase characterized by unique physicochemical properties [95].

Beyond its role in organizing nucleolar structures, LLPS is important for the nucleolus's ability to respond to cellular changes in demand and stress. The fluid nature of phase-separated compartments enables the nucleolus to rapidly assemble and disassemble in response to changes in ribosome production or environmental conditions. For example, under stress, such as DNA damage or inhibition of transcription, nucleolar proteins like nucleophosmin (NPM1) can undergo phase separation in the nucleoplasm, triggering stress response pathways such as p53 signalling [96]. This dynamic behaviour allows the nucleolus to act as a sensor of cellular homeostasis. Additionally, LLPS can facilitate the spatial organization of ribosome biogenesis by concentrating enzymes, substrates and cofactors within specific phases, thereby enhancing the efficiency and fidelity of rRNA processing and ribosome assembly [97]. Overall, the nucleolus’s capacity to form distinct yet interconnected phases through LLPS ensures the coordinated regulation of complex processes and enables rapid responses to cellular signals [95].

## 5. Evolutionary mysteries: prokaryotic compartmentalization and the origins of the nucleolus

### 5.1. Compartmentalization in prokaryotes: membrane-bound or protein-based structures?

While eukaryotes are characterized by their membrane-bound organelles (e.g. nucleus, mitochondria), recent studies reveal a surprising degree of compartmentalization in prokaryotes, both with and without membrane-bound structures. This emerging evidence challenges the traditional view of prokaryotic simplicity and suggests that prokaryotes possess sophisticated mechanisms to organize biochemical processes, akin to those found in eukaryotic cells [98]. Prokaryotic cells exhibit various compartmentalization strategies, ranging from protein-based microcompartments to membrane-bound organelles, offering important insights into the evolutionary origins of cellular complexity.

Bacterial microcompartments (BMCs) are protein shells encapsulating specific enzymes and metabolites, creating localized and specialized environments for metabolic processes. Notably, these structures serve as a prime example of protein-based compartmentalization in prokaryotes. Examples include carboxysomes in cyanobacteria, which are critical for carbon fixation through a CO₂-concentrating mechanism (CCM); these icosahedral structures encapsulate the enzyme RuBisCo along with carbonic anhydrase, enhancing photosynthetic efficiency by concentrating CO_2_ and minimizing photorespiration [99]⁠. Recent studies have revealed the structural diversity of BMCs, with some variants, such as propanediol utilization and ethanolamine utilization microcompartments, playing roles in the metabolisms of organic compounds [100]. The assembly of BMCs is tightly regulated by specific targeting sequences that direct enzymes to the compartment lumen, a process reminiscent of the targeting mechanisms employed by eukaryotic organelles [101].

In addition to protein-based compartments, other specialized structures further illustrate prokaryotic versatility. Gas vesicles have been identified as another example that further illustrates the versatility of prokaryotic compartmentalization. These structures aid buoyancy in aquatic environments through protein assemblies that allow gas diffusion [102]⁠. Additionally, some Planctomycetes, like those in the genus Planctomycetes, contain membrane-bound organelles such as anammoxosomes, which support anaerobic ammonium oxidation essential to the nitrogen cycle. Anammoxosomes are uniquely characterized by their ladderane lipid bilayers, which create a highly impermeable environment necessary for these reactions [103]⁠. Another example is *Gemmata obscuriglobus*, a Planctomycete with a double membrane surrounding its nucleoid, suggesting a level of compartmentalization previously thought to be exclusive to eukaryotes [104]. These findings not only challenge the simplistic view of prokaryotic cells but also hint at an ancestral toolkit of compartmentalization mechanisms that may have contributed to the evolution of membrane-bound organelles, such as the nucleus, in eukaryotes.

### 5.2. The nucleoid and its role in prokaryotic compartmentalization

Another notable structure in prokaryotes is the nucleoid, an irregular, membrane-free region containing most of the genetic material. Although lacking a surrounding membrane, the nucleoid exhibits a high degree of organization and spatial regulation. In some prokaryotes, the nucleoid is positioned at the cell poles, suggesting an organized spatial arrangement that separates it from ribosome-rich areas or riboids [105,106]⁠. This spatial segregation is important for coordinating transcription and translation. In the *Pirellula marina*, a Planctomycete, the nucleoid is enclosed within a structure called the pirellulosome. This compartment not only contains the nucleoid but also includes ribosomes and transcription machinery, resembling the compartmental organization observed in eukaryotic nuclei [107]⁠. Recent studies have revealed that pirellulosome is surrounded by a proteinaceous envelope, which may play a role in maintaining the integrity of this compartment [98]. The presence of these structures in a prokaryote challenges the traditional view of prokaryotic simplicity and suggests that the evolutionary origins of nuclear compartmentalization may be more complex.

Additional examples, such as the membrane-separated structures observed in *Ignicoccus hospitalis* [108] and the double membrane surrounding the nucleoid in *Gemmata obscuriglobus* [109], further emphasize the complexity of prokaryotic cellular organization. Moreover, structures similar to nuclear pores have been observed in Planctomycetes, suggesting that selective molecular trafficking may also exist in these cells [110].

These findings challenge the simplistic view of prokaryotic cells and prompt intriguing questions about the evolutionary origins of eukaryotic cellular components, such as the nucleolus. While membrane-bound compartments are central to eukaryotic cells, there are also membrane-free structures with specialized functions. Studying analogous structures in bacteria and archaea may provide insight into the evolutionary development of the nucleolus and mechanisms for gene expression and ribosome biogenesis in early life forms.

### 5.3. The evolutionary origins of the nucleolus

The eukaryotic nucleolus is a membrane-less subnuclear compartment with distinct structural regions (e.g. fibrillar centre (FC), dense fibrillar component (DFC) and granular component (GC)), specialized in ribosome biogenesis. However, its functions extended beyond this, encompassing critical roles in cellular stress responses, cell cycle regulation and the biogenesis of other ribonucleoprotein particles. Despite its central role its evolutionary origins remain enigmatic, offering a rich study area for evolutionary and cell biologists.

**Figure 3.**
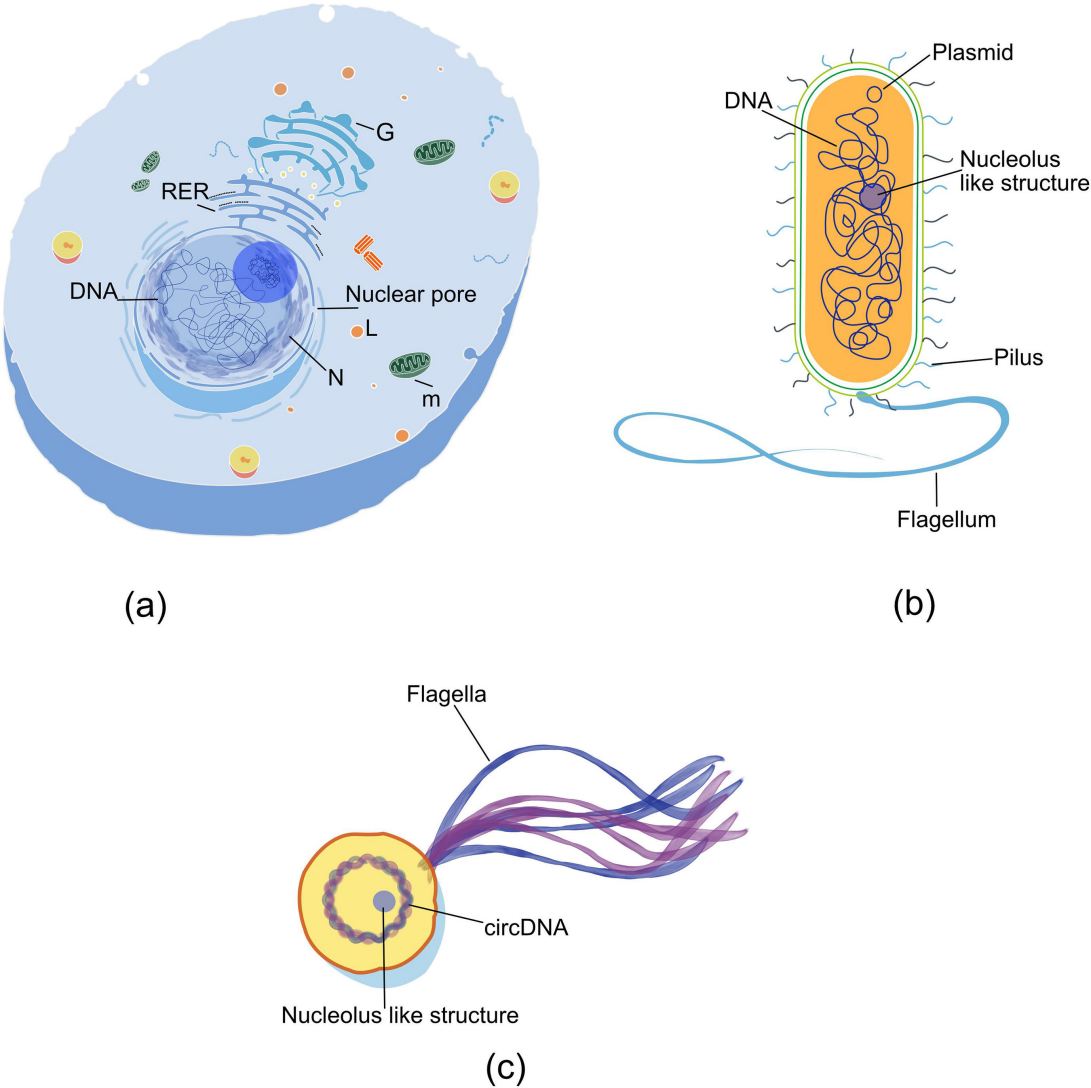
Comparative structures between eukaryotic nucleolus and prokaryotic nucleolus-like components comparative view of eukaryotic and prokaryotic cells, highlighting nucleolus-like structures across different domains of life. (a) The eukaryotic cell displays a distinct nucleolus within the nucleus (n), surrounded by nuclear pores and other cellular components such as mitochondria (m), rough endoplasmic reticulum (RER) and Golgi apparatus (g). (b) In certain bacterial species, a nucleolus-like structure is observed, where DNA and ribosomal RNA components are organized in a central region. (c) In an archaeal cell model, a similar nucleolus-like structure is shown, associated with circular DNA (circDNA) and flagella, hinting at compartmentalization mechanisms in prokaryotes that parallel eukaryotic nucleolar functions.

Comparative studies of prokaryotic and eukaryotic cells provide valuable clues into the nucleolus’ evolutionary trajectory, as some aspects of ribosome biogenesis and gene regulation seen in eukaryotes may have originated in prokaryotes. For example, while prokaryotes lack a true nucleolus, they perform comparable functions within the cytoplasm (figure 3), suggesting that the foundational mechanisms of ribosome biogenesis and gene regulation may have originated in prokaryotic ancestors. For instance, the presence of homologous proteins, such as fibrillarin and small nucleolar RNAs (snoRNAs) found in archaea, hint at an evolutionary link to eukaryotic nucleolar functions, possibly tracing back to the last eukaryotic common ancestor (LECA) [111]⁠. The conservation of these proteins across domains of life underscores the importance of ribosome biogenesis as a fundamental cellular process, one that has been refined and compartmentalized over billions of years of evolution.

A detailed comparison of nucleolar proteins in eukaryotes and their functional analogs in prokaryotes (table 2) reveals a global vision of the conserved mechanisms and evolutionary connections shared among different life forms. These comparisons highlight how certain core processes, such as rRNA processing and ribosome assembly, have been preserved across evolutionary time, even as the cellular structures housing these processes have diverged significantly. This conservation suggests that the nucleolus, as we know it in eukaryotes, may have evolved through the gradual specialization and compartmentalization of pre-existing prokaryotic functions rather than through *de novo* emergence of entirely new mechanisms.

**Table 2.** Some nucleolar molecules in eukaryotes and prokaryotes.

eukaryotic protein or RNA molecule	**f**unction in eEukaryotes	homologue/analogue in prokaryotes	function in prokaryotes	similarities	references
fibrillarin	rRNA modification (methylation, transcription and pseudouridylation) in the DFC	archaeal fibrillarin, Cbf5	rRNA modification in archaea	both have a methyltransferase domain and participate in rRNA maturation	[31,112,113]
nucleophosmin (NPM1/B23)	ribosome assembly, cell cycle regulation, DNA repair, stress response and genomic stability	no direct homologue, but proteins like RbfA, RsgA, ERA and ObgE have similar roles	ribosome assembly, stress response and rRNA processing	both are involved in ribosome assembly, stress response and rRNA processing	[114–120]
nucleolin	rRNA processing, cell growth, synthesis of ribosomes and transcription regulation	no direct homologue, but proteins like Hfq and CsrA regulate RNA and may have analogous roles	RNA regulation and processing	both are involved in RNA processing and regulation	[121–124]
snoRNAs	guide chemical modifications of rRNA (methylation and pseudouridylation)	sRNAs (small RNAs)	gene expression regulation and RNA processing	both are small, non-coding RNAs that interact with proteins to modify or regulate RNA	[125–128]
RNA polymerase I	transcription of rRNA in the nucleolus and ribosomal biogenesis	RNA polymerase (bacteria/archaea)	transcription of rRNA in prokaryotes	both are responsible for rRNA synthesis, though eukaryotic Pol I is specialized for rRNA transcription	[129–131]
pescadillo (PES1)	pre-rRNA processing, ribosome biogenesis and DNA replication	no direct homologue, but proteins like RimM and RbfA participate in rRNA processing and ribosome assembly	rRNA processing and ribosome assembly	both are involved in rRNA processing and ribosome assembly	[132–134]
UTP (U3 snoRNA-associated proteins)	pre-rRNA processing and ribosome biogenesis	no direct homologue, but proteins like NusB and NusG are involved in rRNA transcription and processing	rRNA transcription and processing	both are involved in rRNA processing and transcription regulation	[135–140]
nopp140	ribosome biogenesis and nucleolar organization	no direct homologue, but proteins like NusA and NusG are involved in transcription regulation	transcription regulation and RNA processing	both are involved in transcription regulation and RNA processing	[141,142]
RRN3 (Transcription Initiation Factor)	required for RNA polymerase I transcription initiation	no direct homologue, but proteins like sigma factors (σ70) regulate transcription initiation	transcription initiation	both are involved in transcription initiation	[143–146]
nop56/Nop58	rRNA modification and snoRNA binding	archaeal Nop56/Nop58 homologues	rRNA modification in archaea	both are involved in rRNA modification and interact with snoRNAs/sRNAs	[147–150]
RPL5 (Ribosomal Protein L5)	ribosome assembly and 5 S rRNA binding	ribosomal protein L18 in prokaryotes	ribosome assembly and 5 S rRNA binding	both are ribosomal proteins involved in rRNA binding and ribosome assembly	[151–157]
RPL11 (ribosomal protein L11)	ribosome assembly and p53 regulation	ribosomal protein L11 in prokaryotes	ribosome assembly and stress response	both are ribosomal proteins involved in ribosome assembly and stress signalling	[158–161]

The study of early-branching eukaryotes, such as *Giardia lamblia*, provides further clues to the nucleolus’s evolutionary history. These organisms contain simplified nucleolar-like regions, which, while less complex than those in late divergent eukaryotes, still perform essential nucleolar functions. This suggest that the nucleolus may have evolved differently across eukaryotic lineages [162], adapting to the specific needs and constraints of each lineage⁠.

**Figure 4.**
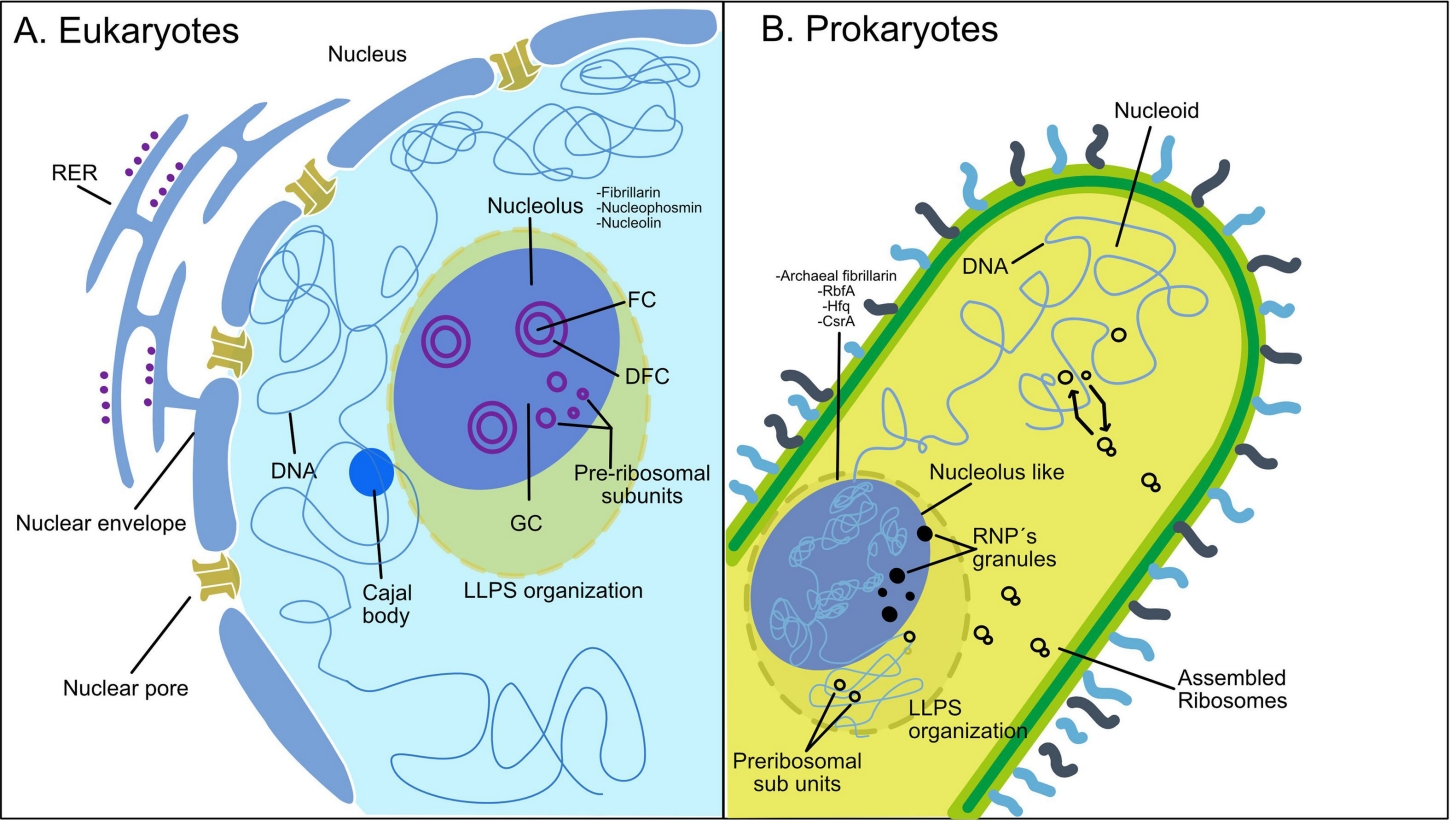
Detailed features of eukaryotic nucleolus and prokaryotic nucleolus-like structures. Comparative overview of the structural elements found in the nucleolus of eukaryotic cells and the nucleolus-like structures in prokaryotic organisms. Panel (A) illustrates a section of the eukaryotic nucleolus, highlighting its key components. The fibrillar centre (FC) in which ribosomal RNA (rRNA) transcription occurs, dense fibrillar centre (DFC) contains newly synthesized rRNA along with some proteins essential for ribosomal assembly. Surrounding these is the granular component (GC), where pre-ribosomal particles are formed. The diagram also indicates a Cajal body, which plays a role in RNA processing and assembly; the diagram also indicates the DNA, nuclear envelope and nuclear pores. Important proteins such as fibrillarin, nucleophosmin and nucleolin are listedto underscore their roles in ribosome biogenesis. Additionally, liquid–liquid phase separation (LLPS) is highlighted as an essential process for nucleolus organization. Panel (B) depicts a prokaryotic cell, showing its nucleolus-like structures that serve similar functions in ribosome assembly. The diagram identifies the nucleoid, which contains prokaryotic DNA, alongside various components such as RNP granules, pre-ribosomal subunits and assembled ribosomes. Key proteins involved in these processes are indicated, including archaeal fibrillarin, RbfA, Hfq and CsrA, which facilitate RNA processing and ribosomal function in prokaryotes. Additionally, the role of LLPS is highlighted as a significant mechanism allowing the formation of distinct biochemical compartments within the cell that may contribute to the organization of nucleolus-like structures in prokaryotes.

The compartmentalization of cellular processes is linked to the emergence and specialization of the nucleolus; this functional differentiation allowed the nucleolus to specialize in rRNA processing and ribosome assembly, functions that are distinct from other nuclear activities (figure 4A). This spatial and functional segregation likely provided a selective advantage, allowing for more efficient and regulated ribosome production.

Comparative studies of nucleolar proteomes between early and later-diverging eukaryotes reveal that many essential nucleolar proteins are conserved, indicating that ribosome-related functions were primordial to nucleolar evolution. For example, in *Prorocentrum micans*, a dinoflagellate, there is a ‘persistent’ nucleolus that remains intact during cell division, in contrast to the ‘autonomous’ nucleoli in other dinoflagellate species that disassemble and reassemble during cell division [163]. This persistent nucleolus is intriguing because it suggests a unique interaction between nucleolar elements and chromosomes during cell division. Moreover, this feature is shared with some plant species [164]⁠ and may be significant in terms of ribosome biogenesis and cellular efficiency.

Some organisms like *Trypanosoma brucei* exhibit a single nucleolus, which lacks fibrillar centres (FCs) found in another nucleolus; instead, they have a slightly dense fibrillar component (DFC). The absence of the FC may be correlated with a relatively low number of rRNA gene copies (with only one or two dozen) present in trypanosomes, compared with the hundreds present in higher eukaryotes [165]⁠.

Comparative studies of prokaryotic and eukaryotic ribosome biogenesis also highlight evolutionary links. In bacteria, ribosome assembly involves the transcription of precursor rRNAs (23S, 16S and 5 S) and the stepwise assembly of ribosomal subunits in the cytoplasm. This highly efficient process, which produces up to 100 000 ribosomes per hour, relies on assembly factors that facilitate RNA folding and protein binding under diverse conditions [166,167]⁠; this process is highly efficient and facilitated by assembly factors that guide RNA folding and protein binding for ensuring correct ribosome formation under different cellular conditions [168]⁠. Although less understood in archaea, ribosome biogenesis shares similar features with bacteria, albeit with unique proteins and assembly pathways. Members of the TACK Archaea group possess nucleolar protein homologues, such as fibrillarin, suggesting an evolutionary link with eukaryotic nucleolar functions [169]⁠.

Some bacterial species also exhibit nucleolus-like compartmentalization for the transcription process; co-localization experiments of RNA polymerase and ribosomal RNA operons suggest a spatial organization to resemble eukaryotic nucleoli organization inner nuclei (figure 4), which is evidence for the strategies for efficient transcription and translation optimizing cellular functions despite the absence of membrane-bound compartments like nuclei in eukaryotes [170]⁠.

The principles of LLPS provide a compelling framework for understanding the evolutionary origins of the nucleolus as a membrane-less structure. The formation of these type of compartments through phase separation likely provided an early mechanism for organizing biochemical processes in primitive cells. Prokaryotes exhibit ribonucleoprotein (RNP) granules, that its formation is through LLPS and perform functions similar to those of the nucleolus [171]. From an evolutionary perspective, the nucleolus can be viewed as a product of the co-option of LLPS mechanisms that were already present in early life forms, allowing for the efficient organization of ribosome biogenesis in response to the increasing demands of eukaryotic cells [95]. The presence of nucleolar-like structures in early branching eukaryotes, such as *Giardia lamblia*, further supports this idea, highlighting the evolutionary conservation of LLPS in nucleolar function. Moreover, studies in model organisms like *Drosophila melanogaster* have demonstrated the dynamic nature of LLPS in nucleolar proteins, underscoring its importance in maintaining nucleolar integrity and function [172]. Thus, LLPS not only explains the dynamic organization of the modern nucleolus but also provides a framework for understanding its evolutionary development.

The nucleolus is not merely a static structure; rather, it is a dynamic and fundamentally evolutionary entity. Its origins may lie in the deep past, rooted in the molecular machinery of prokaryotic ancestors, and its evolution has shaped cellular compartmentalization, specialization and functional diversification. Further exploration of archaeal ribosome biogenesis and the spatial organization of transcription and translation in bacteria may shed light on the evolutionary origins of the nucleolus. Such studies hold the potential to deepen our understanding of nucleolar specialization in early-diverging eukaryotes and may reveal how prokaryotic cells developed the complex cellular architectures that later contributed to eukaryotic complexity.

## 6. The significance of studying the nucleolus: evolutionary origins and functions

### 6.1. Uncovering the evolutionary origins of the nucleolus

The nucleolus has long intrigued researchers due to its central role in ribosome biogenesis and broader cellular functions. Although membrane-bound organelles are unique to eukaryotes, intriguing similarities between the nucleolus and certain prokaryotic structures suggest a shared evolutionary history. For example, some bacteria show RNA polymerase clustering at rRNA operons, forming ‘transcriptional factories’ that resemble the organization seen in the eukaryotic nucleolus, optimizing ribosome biogenesis under favourable conditions [170]⁠.

As we mentioned earlier, some research suggests that specific proto-nucleolar structures may exist in some archaea, hinting at a possible evolutionary precursor to the nucleolus; however, these structures are still largely unknown, with many aspects still to be investigated. Notably, specific protein domains found in nucleolar proteins are present in the three domains of life, indicating that they form part of the repertoire of the last universal common ancestor (LUCA) [171]⁠. Additionally, evidence suggests that more protein domains associated with eukaryotic nucleolus are detectable in archaea than in bacteria. Protein domains that suggest implications in ribosomal biogenesis hinting at an archaeal ancestry for core nucleolar proteins [173]⁠. Recent studies have provided intriguing insights into the presence of nucleolus-like structures in some representants of archaea, such as *Sulfolobus solfataricus*, which possess homologues of nucleolar proteins and exhibit ultrastructural features similar to those of eukaryotic nucleoli [169]⁠, putting in mind that the evolutionary origins of the nucleolus may be more complex than previously thought, potentially involving a shared ancestry with specific prokaryotic lineages. Moreover, discussions have emerged regarding the absence of detectable nucleolus in prokaryotes and how some proteins are essential for maintaining nucleolar integrity [174]⁠.

Many open questions still need to be addressed, and we expect discoveries to arise; these findings will likely be significant in shaping and refining our current understanding of the nucleolus, shedding light on its evolutionary origins and relationships, diversity of functions and role in cellular function. This ongoing exploration is vital not only for comprehending the nucleolus itself but also for understanding potential implications for human health since dysfunctions of the nucleolus have been linked to various diseases. Releasing fundamental biological principles of nucleolus evolution could lead to innovative therapeutic strategies.

### 6.2. Medical significance of nucleolar research

The study of nucleolar function has implications beyond evolution, especially in understanding human health and disease. The nucleolus is central to ribosome biogenesis, which directly affects protein synthesis, cell growth and proliferation. Abnormalities in nucleolar function and structure are linked to various diseases, including ribosomopathies, genetic disorders resulting from mutations in genes encoding ribosomal components or biogenesis factors [175,176]⁠. This connection between ribosome production and cellular function makes the nucleolus a significant focus in studying cell-cycle regulation and cancer [177]⁠.

Nucleolar dysfunction has been associated with ageing and complex diseases such as progeria, cancer and neurodegenerative disorders [178]⁠. Morphological changes in the nucleolus observed in these conditions indicate potential roles for non-canonical nucleolar functions beyond ribosome biogenesis. Exploring these non-canonical functions could yield insights into disease mechanisms and provide targets for personalized medical therapies. For example, understanding the nucleolus’s role in stress responses and cellular regulation could lead to innovative treatments aimed at modulating nucleolar activity.

Despite considerable progress, many questions about the nucleolus’s role in health and disease remain open. Ongoing research promises to deepen our understanding of nucleolar structure and function, potentially revealing new avenues for therapeutic strategies that target nucleolar functions to treat diseases linked to its dysfunction.

## 7. Conclusions: the nucleolus in focus

Ongoing research into the nucleolus and its evolutionary origins is essential for addressing unanswered questions about this structure and its potential clinical implications [179]⁠. This review has explored the nucleolus from structural, functional and evolutionary perspectives, highlighting its role not only in ribosome biogenesis but also in broader cellular functions [180]⁠. Understanding the nucleolus helps enrich our grasp of cellular biology and opens pathways for investigating lesser-known aspects of this intriguing, and in some organisms, elusive substructure [99]⁠.

As new labelling, molecular and imaging technologies continue to advance, they allow for unprecedented insights into the nucleolus’s dynamic nature [102,181]⁠. While canonical roles, such as its involvement in the cell cycle and ribosome synthesis, are well established, there is growing interest in the nucleolus’s non-canonical functions, which remain largely unexplored [182]⁠. Expanding our study of both canonical and non-canonical functions could deepen our understanding of cell biology and provide new avenues for therapeutic innovation [178]⁠.

Addressing open questions surrounding nucleolar function and evolution also highlights the nucleolus’s critical role in maintaining cellular homeostasis [26]⁠. Exploring its structural and functional diversity across different lineages may uncover fundamental biological principles that link the nucleolus to cellular complexity and adaptability [107]⁠. Ultimately, the future of nucleolar research holds promise for advancing our knowledge of cellular function, disease mechanisms and novel therapeutic strategies that could impact significant health challenges [9,10]⁠.

## Data Availability

This article has no additional data.
